# The Potential for Neuromodulation in the Treatment of Alzheimer’s Disease: A Review of Clinical Trials

**DOI:** 10.7759/cureus.85156

**Published:** 2025-05-31

**Authors:** Trevor Jones, Moshe Shalom, Anjalika Chalamgari, Justin Gold, Brolyn Zomalan, Saarang Patel, Vikas Munjal, Mohammad F Khan, Yuncong Mao, Julian L Gendreau, Mickey E Abraham

**Affiliations:** 1 Department of Neurosurgery, University of California San Diego, San Diego, USA; 2 Department of Neurosurgery, Children’s Hospital at Montefiore, Bronx, USA; 3 Department of Surgery, University of Florida, Gainesville, USA; 4 Department of Medicine, Rowan University Cooper Medical School, Camden, USA; 5 Department of Neurosurgery, Seton Hall University, South Orange, USA; 6 Department of Neurosurgery, Ohio State University College of Medicine, Columbus, USA; 7 Department of Neurosurgery, Indiana University School of Medicine, Indianapolis, USA; 8 Department of Neurosurgery, Johns Hopkins University School of Medicine, Baltimore, USA; 9 Department of Biomedical Engineering, Johns Hopkins University, Baltimore, USA

**Keywords:** alzheimer’s disease, clinical trials, neuromodulation, tdcs, tms

## Abstract

There is still no cure for Alzheimer’s disease (AD), which remains the leading cause of dementia in Western countries. Neuromodulation, the use of electrical or chemical interventions to modify neuronal excitability, has shown promise in treating several neurological conditions and has become a topic of interest in the context of AD. We aim to review clinical trials related to neuromodulation in AD.

Analysis of current clinical trials was conducted using ClinicalTrials.gov. The search term used was “Alzheimer’s disease,” and results were filtered for studies that included neuromodulation.

One hundred and eleven clinical trials were found, and 82 trials remained after exclusion. All trials utilized some form of neuromodulation device as the primary intervention, with transcranial magnetic stimulation and transcranial direct current stimulation as the most common modalities. Thirty-six (43.9%) trials were completed, 20 (24.3%) were not yet recruiting, 23 (28.0%) were actively recruiting, and three (3.7%) were enrolling by invitation. Of the completed trials, only 11 (30.6%) had associated results, and of those 11, eight (22.2% of completed trials, 72.7% of trials with results) were associated with published articles in a peer-reviewed journal. All but one of the eight trials displayed some form of improvement in their metric of choice.

Although the number of trials with published results is limited, there appears to be positive evidence of the efficacy of neuromodulation in treating AD. The medical community must continue to emphasize the need for additional clinical trials in this area.

## Introduction and background

In 1906, Dr. Alois Alzheimer described an unknown condition in one of his patients characterized by behavioral changes, persistent confusion, and memory loss. Postmortem histology of the brain tissue from this patient revealed clumps and tangled fibers, which the medical community would later recognize as amyloid plaques and Tau tangles, respectively. These proteins, which are normally benign and aid in neuronal structural support, become misfolded and induce misfolding in other amyloid and tau proteins [1]. The spreading of these misfolded proteins throughout the cortex leads to neuronal atrophy and symptoms similar to those reported by Dr. Alois Alzheimer [2]. This disease has become known to the medical community as Alzheimer’s disease (AD) and has become an important component of both clinical practice and medical research.

Currently, AD is the most common cause of dementia in Western countries, affecting 5.5 million individuals in the United States and 24 million worldwide [3]. The incidence of AD increases with age, with rates beginning at 17% at the age of 60 and doubling every five years [4]. Despite this, the causes of AD are still largely unknown, and it seems as though genetic and lifestyle factors play important roles in its pathogenesis. Cerebrovascular disease, along with behaviors and conditions that lead to it, has been shown to frequently coexist with AD [5]. Genetic predisposition to AD also exists, largely with regard to the apolipoprotein E (APOE) gene. Approximately 20% of individuals living with AD have the Epsilon-4 genotype of APOE [6].

Dementia is the most prominent symptom of AD and progresses as levels of neurofibrillary tangles in the medial temporal lobe increase [7]. The amount of misfolded tau protein more closely correlates with the severity of the disease. Amyloid beta plaques also increase with disease progression but tend to be less localized to the cortex [8]. Traditionally, treatment strategies against AD have been limited and largely drug-based. Although current drug therapies have been shown to decrease symptoms and reduce dementia in some patients, a therapy that halts the disease process has yet to be discovered [9].

Neuromodulation, the use of electrical and chemical interventions to modify neuronal activity, has proven beneficial in the treatment of neurological and psychiatric conditions. For example, transcranial magnetic stimulation (TMS), a process that induces or inhibits electrical currents in cortical tissue using extracranial magnetic fields, has already been established as a treatment for depression [10]. Although the mechanism by which TMS relieves patients of depression remains unclear, it has been suggested that its benefits can be attributed to increased synaptic plasticity [11]. As neuronal death is a hallmark of AD, it has been postulated that neuromodulatory effects on synaptic plasticity may be beneficial in the treatment of AD. Additionally, there are some estimates that up to 19% of those with dementia have concurrent depression [12]. It has also been found that depression is a risk factor for the development of dementia, with an increased risk of 50% of developing dementia over a 17-year period in individuals who have had depression [13].

The lack of a definitive cure for AD, along with the advent of neuromodulation in the treatment of neurological conditions, prompts us to ask whether or not neuromodulatory strategies are the next step in the search for a cure to AD. Clinical trials bridge the gap between the laboratory and the clinic and are crucial in the application of new treatment modalities to patient care [14]. The primary objective of the present study is to describe the current status of clinical trials that utilize neuromodulatory techniques in the treatment of AD and to emphasize the importance of continuing research on this topic. Secondary objectives include an analysis of the included clinical trials with regard to individual study characteristics, such as the devices used or the diagnoses studied. We hope that this analysis may highlight what is presently being studied and offer insight into the current state of the field.

## Review

Methods

Database Search

A thorough search of ClinicalTrials.gov was conducted using the search term "Alzheimer's disease." Trials were then filtered to include only those that involved neuromodulating devices. Clinical trials that had been suspended, terminated, withdrawn, or had an unknown status were excluded from the present study.

Of the trials that met the described inclusion criteria, data on trial status, study title, conditions, interventions, study type, study design, outcome measures, number enrolled, age of participants, National Clinical Trial (NCT) number, study start/study completion date, and location were collected and tabulated in the Appendices section. For those trials that were completed with results, an attempt to retrieve those results was made on September 13, 2022, using PubMed.gov.

Statistical Analysis

Descriptive statistics were used for the initial assessment. Pearson's chi-squared correlational coefficient testing was used to assess any trends in clinical trials over subsequent years.

Results

Search Results

**Figure 1:**
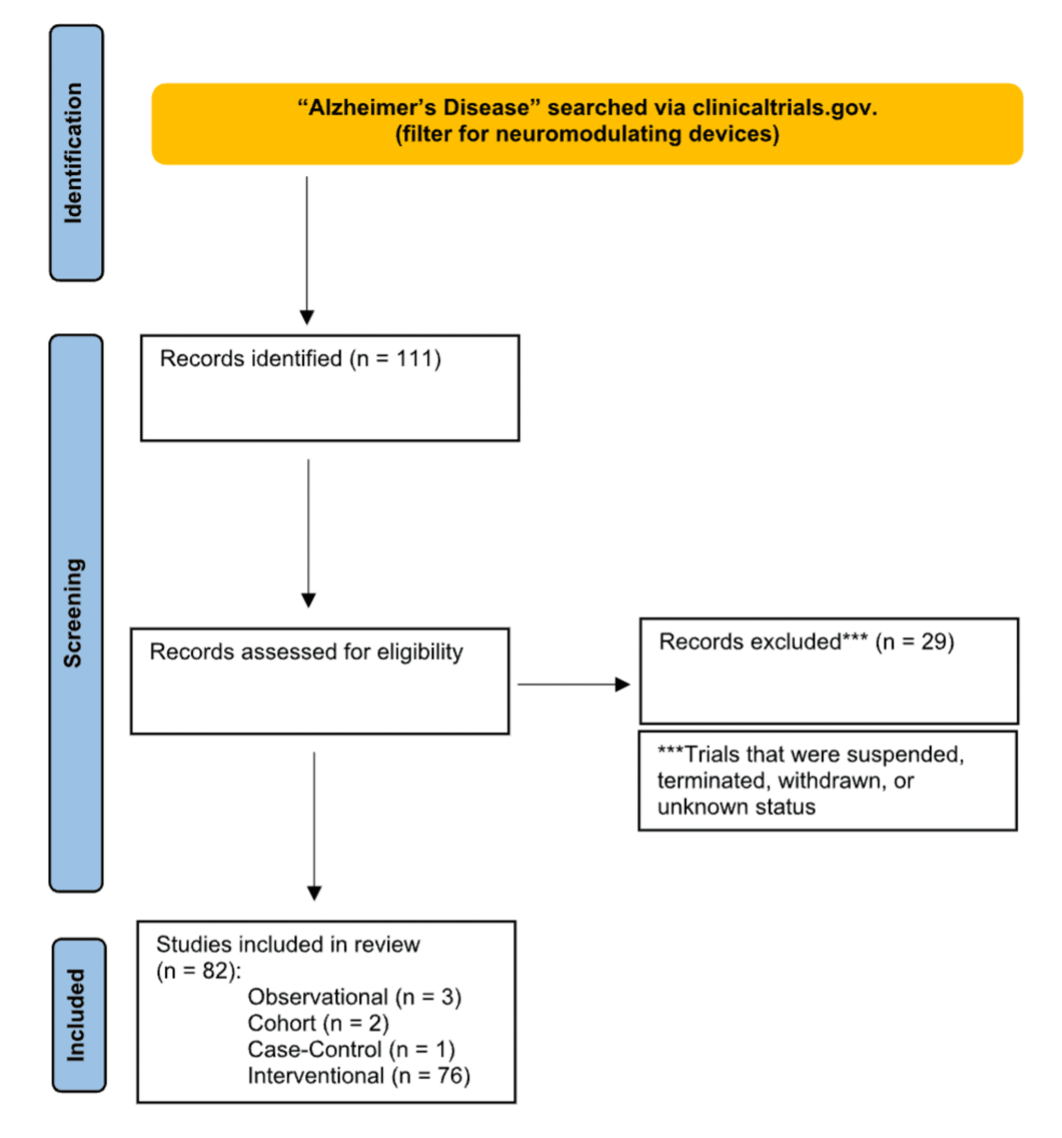
Diagram showing the studies identified, screened, and included in the review All studies were obtained from ClinicalTrials.gov. Major exclusion criteria included studies that were terminated, withdrawn, suspended, or of unknown status.

Of the 111 trials found based on our search terms, 82 were selected for further analysis based on selection criteria (Figure 1). The Appendices section provides a compilation of the 82 trials, including their associated NCT numbers, the major device used, and enrollment numbers.

Study Characteristics

Study start dates ranged from 2003 through 2022, with 41 (50%) beginning between 2019 and 2022. Thirty-six (43.9%) trials were completed, 20 (24.3%) were not yet recruiting, 23 (28.0%) were actively recruiting, and three (3.7%) were enrolling by invitation.

The trial location varied significantly, with 35 (42.7%) of the 82 trials conducted in the United States and the rest spread throughout Europe, Asia, and, to a lesser extent, the Middle East and Africa. The sample size showed fewer variations, with 57 (69.5%) having a sample size of between 0 and 50 participants, 15 trials (18.3%) between 51 and 100, and 10 (12.2%) trials between 101 and 500. The median sample size was 40 participants.

Age was also relatively standard, with six trials (7.3%) investigating patients 18 years and older and 72 (87.8%) requiring a minimum age of 40. The remaining four had a minimum age requirement ranging from 20 to 35 years. The maximum age was typically between 80 and 90 years or was not set.

Diagnoses

All trials presented here have documented AD as the primary condition, with three trials (3.7%) specifying early-onset AD. Non-AD-related dementias that were included in trials included aging (5, 6.1%), mild cognitive impairment (19, 23.2%), dementia with Lewy bodies (2, 2.4%), frontotemporal dementia (3, 3.6%), Parkinson's disease (1, 1.6%), and nondescript dementia (6, 7.3%). All other trials listed AD as the only studied condition.

Devices

All trials utilized some form of neuromodulation device as the primary intervention. TMS and transcranial direct current stimulation (tDCS) were the most commonly used neuromodulation modalities in 30 trials (36.6%) each. Of the remaining trials, 11 (13.4%) used transcranial alternating current stimulation (tACS), four (4.9%) used electromagnetic wave stimulation, two (2.4%) used nondescript electrical stimulation, and five (61.0%) used deep brain stimulation (DBS).

Measures of change in condition were heavily varied between trials, although all used at least one psychological metric. 49 (59.8%) trials exclusively used psychological testing, and the remaining 33 (40.2%) used a mixture of psychological and neurophysiological testing. Among the trials that employed mixed testing, the most common testing strategies included functional magnetic resonance imaging (fMRI), electroencephalography (EEG), positron emission tomography (PET), or a combination of these tests and others.

Of the completed trials, only 11 (30.6%) had associated results, and of those 11, seven (19.4% of completed trials, 63.6% of trials with results) were associated with published articles in a peer-reviewed journal.

Peer-Reviewed Publications

Only seven trials had published, peer-reviewed results. Brem et al. demonstrated the efficacy of repetitive TMS combined with cognitive training in significantly improving cognition in AD patients [15]. tACS stimulation in AD patients has alternatively been shown to increase blood perfusion to important brain areas [16]. In two separate studies, tDCS was found to increase GABA levels and enhance functional connectivity within neural networks, respectively [17,18]. Interestingly, tDCS was shown to have little effect on ameliorating apathy in AD [19]. Patients undergoing DBS demonstrated better glucose metabolism and performance on cognitive tasks [20,21]. Overall, all but one of the seven trials displayed some form of improvement in their metric of choice.

Discussion

The rapid progress of technological advances in the medical domain continues to prompt the medical community to reevaluate our treatment strategies and first-line therapies for nearly all disease types. This has been ever more evident in neurological conditions such as AD that have thus far been thought to be incurable and prompts us to conduct further research and clinical studies on potential avenues for treatment of these diseases. Neuromodulation has demonstrated potential in treating various neurological conditions and has become an increasingly common clinical tool. Our results indicate that the increasing number of clinical trials initiated each year reflects this interest in extending the spectrum of diseases for which neuromodulation can alleviate symptoms.

The large variation in physiological metrics reflects the diverse range of diagnostic equipment and techniques used for AD. MRI testing was included to check for structural changes, particularly the volume of neuronal atrophy [22]. fMRI and EEG were primarily used to assess changes in connectivity. TMS, while also a treatment, was used to validate tDCS and tACS, indicating a change in excitability. CSF and blood checks were used as a means of indicating the presence of AD biomarkers, predominantly amyloid beta plaques. Likewise, PET scans were used to check for amyloid beta plaques in the brain [23]. Finally, arterial spin labeling was used to check tissue perfusion of the affected area.

While pharmacological treatments for AD may not disappear, the increase in the number of clinical trials testing neuromodulation in this context offers hope that other avenues of treatment will soon become available for patients, especially those for whom pharmacological therapy has been insufficient. Albeit the growing interest in neuromodulation, only a small percentage of the trials presented here have been completed, likely due in part to delays from the COVID-19 pandemic and limited accessibility to neuromodulation devices. Despite this, however, we will likely not have to wait long for further results, as most trials that have begun recently have a completion window of two to four years.

For trials that have been completed, results are promising. Although the exact mechanism by which AD leads to cognitive decline remains to be elucidated, considerable discussion has been devoted to this topic. One leading hypothesis suggests that the amyloid deposits characteristic of AD inhibit synaptic function and lead to a decrease in dendritic spine density [24]. We believe, as has been suggested previously [25], that neuromodulation may alleviate the cognitive decline associated with AD via its effects on long-term potentiation. It has been demonstrated that neurostimulation can evoke long-term potentiation, a phenomenon similar to physiological neuroplasticity [26]. This line of thought is also consistent with the notion presented in several trials that earlier intervention during disease development may lead to better patient outcomes. Due to the atrophy associated with AD, there comes a point in disease progression when there is too much atrophy and not enough functioning neuronal tissue to be stimulated [27]. This similarly supports our hypothesis, as long-term potentiation can only occur in live neurons.

Although neuromodulation shows promise in treating AD, it is likely to only slow disease progression, similar to the current treatment paradigm. While the inductive changes that rogue protein structures create will likely not be halted by the increased activation of the areas they are destroying, we predict that this treatment modality will improve quality of life by providing an alternative to pharmacological treatment, which may not work in every patient and may lead to unwanted side effects [28].

The published results from our highlighted trials already demonstrate that this prediction is true. TMS, for example, has shown its efficacy in significantly improving cognition relative to control [15]. tACS dramatically increased blood perfusion to the temporal lobe in one study [16]. Other neuromodulatory methods, like tDCS, have even been shown to replicate pharmacological effects by modifying neurotransmitter levels in the brain [17]. tDCS also improved performance on memory tasks by almost 7% [18]. While further research is needed to elucidate the potential benefits of incorporating neuromodulatory strategies into the AD treatment regimen, it is clear that this work could have significant benefits for patients. The medical and scientific community must emphasize the progress and ultimate completion of clinical trials in this domain so that these strategies can be implemented clinically and improve the quality of care.

## Conclusions

Neuromodulation has become more and more prevalent in the treatment of neurological diseases, and, unsurprisingly, it has become a topic of interest in the treatment of AD. Although there is still a long way to go regarding the completion of current clinical trials, currently available results suggest that neuromodulatory strategies, such as TMS, tDCS, DBS, and others, have the potential to improve the quality of life for AD patients.

## Appendices

**Table 1:** Compilation of included studies (n=82) with NCT number, device utilized, and number of participants ITB: intermittent theta burst stimulation, TBS: theta burst stimulation, NCT: National Clinical Trial, DBS: deep brain stimulation, GENUS: gamma entrainment using sensory stimuli, rTMS: repetitive transcranial magnetic stimulation, tACS: transcranial alternating current stimulation, tDCS: transcranial direct current stimulation, TMS: transcranial magnetic stimulation, HD-tDCS: high-definition transcranial direct current stimulation

NCT number	Intervention	Enrollment
NCT05292222	ITB	10
NCT01559220	DBS	3
NCT04042922	GENUS device	80
NCT04562506	rTMS with H-coil	30
NCT04055376	GENUS device	15
NCT04785053	tACS	34
NCT04856072	DBS	12
NCT03288363	tDCS	80
NCT03920826	tACS	46
NCT03121066	TMS	45
NCT05251649	tACS with 40 Hz sound stimulation	60
NCT04088643	tACS	40
NCT03612622	TMS	47
NCT05389644	ITB	20
NCT04771845	TMS	73
NCT04333329	NEUROLITH device	15
NCT01094145	DBS	6
NCT03880240	tACS	55
NCT05643326	tACS	30
NCT04042532	TBS	70
NCT01179373	TMS	45
NCT01847586	Paired associative stimulation	49
NCT03412604	tACS	5
NCT00814697	rTMS	12
NCT01481558	tDCS	40
NCT05575271	Vagus nerve stimulation	6
NCT01504958	rTMS	22
NCT01481961	rTMS	15
NCT05508841	tDCS	42
NCT04823819	Magnetic stimulator DuoMag device	40
NCT03290326	tACS	17
NCT05454540	rTMS	60
NCT03778151	rTMS	50
NCT04783350	tACS	20
NCT05509387	tDCS	42
NCT02537496	rTMS	36
NCT05468268	rTMS	40
NCT04555941	ITB	54
NCT04908358	Vagus nerve stimulation	140
NCT05102045	rTMS	27
NCT03770182	NEUROLITH device	60
NCT05655195	GENUS device	50
NCT05206305	GammaSense stimulation device	20
NCT05544201	Transcranial current stimulation	99
NCT01825330	TMS	131
NCT04045990	rTMS	11
NCT04482179	TMS	30
NCT01958437	tDCS	44
NCT04759092	tDCS	8
NCT01746498	tDCS	33
NCT03325205	Anodal tDCS	19
NCT03543878	Flicker	10
NCT03422250	tDCS	45
NCT04265378	tDCS	46
NCT00909285	TMS	20
NCT04599764	HD-tDCS	40
NCT04122001	tDCS	60
NCT04457973	tDCS	40
NCT05216315	tDCS	60
NCT04507815	tDCS	50
NCT04754152	TMS	45
NCT04866979	TBS	200
NCT00658125	DBS	6
NCT05715866	Nesa device	30
NCT03347084	Excitation device	8
NCT04277104	Closed-loop acoustic stimulation	72
NCT05270408	tDCS	500
NCT05032482	Excitation device	45
NCT02155946	tDCS	107
NCT04549155	TMS	40
NCT03622905	DBS	210
NCT01608061	DBS	42
NCT04294888	rTMS	40
NCT05561205	rTMS	8
NCT02190084	rTMS	20
NCT01334450	TMS	10
NCT04842955	tACS	60
NCT03670615	tDCS	60
NCT04515433	tACS	20
NCT01168245	NICE-system device	15
NCT03351452	tDCS	28
NCT04289402	tDCS	24
